# Modeling-Based Estimate of the Vaccination Rate, Lockdown Rules and COVID-19

**DOI:** 10.3390/healthcare9101245

**Published:** 2021-09-22

**Authors:** Chinlin Guo, Wei-Chiao Chang

**Affiliations:** 1 Institute of Physics, Academia Sinica, Taipei 11579, Taiwan; 2 Department of Clinical Pharmacy, School of Pharmacy, Taipei Medical University, Taipei 11031, Taiwan; 3 Center for Regional Anesthesia and Pain Medicine (CRAPM) of Wan Fang Hospital, Taipei Medical University, Taipei 11696, Taiwan; 4 Department of Pharmacy, Wan Fang Hospital, Taipei Medical University, Taipei 11696, Taiwan

**Keywords:** COVID-19, vaccination rate, basic reproduction number, lockdown

## Abstract

COVID-19 has become a severe infectious disease and has caused high morbidity and mortality worldwide. Restriction rules such as quarantine and city lockdown have been implemented to mitigate the spread of infection, leading to significant economic impacts. Fortunately, development and inoculation of COVID-19 vaccines are being conducted on an unprecedented scale. The effectiveness of vaccines raises a hope that city lockdown might not be necessary in the presence of ongoing vaccination, thereby minimizing economic loss. The question, however, is how fast and what type of vaccines should be inoculated to control the disease without limiting economic activity. Here, we set up a simulation scenario of COVID-19 outbreak in a modest city with a population of 2.5 million. The basic reproduction number (*R*_0_) was ranging from 1.0 to 5.5. Vaccination rates at 1000/day, 10,000/day and 100,000/day with two types of vaccine (effectiveness *v* = 51% and 89%) were given. The results indicated that *R*_0_ was a critical factor. Neither high vaccination rate (10,000 persons/day) nor high-end vaccine (*v* = 89%) could control the disease when the scenario was at *R*_0_ = 5.5. Unless an extremely high vaccination rate was given (>4% of the entire population/per day), no significant difference was found between two types of vaccine. With the population scaled to 25 million, the required vaccination rate was >1,000,000/day, a quite unrealistic number. Nevertheless, with a slight reduction of *R*_0_ from 5 to 3.5, a significant impact of vaccine inoculation on disease control was observed. Thus, our study raised the importance of estimating transmission dynamics of COVID-19 in a city before determining the subsequent policy.

## 1. Introduction

The SARS-CoV-2 (COVID-19) pandemic has caused huge economic losses by the great lockdown. As of May 2021, ~171 million cases with 3.6 million deaths had been reported [1]. SARS-CoV-2 spreads rapidly despite its lower mortality rate, being far more transmissible than SARS-CoV in 2002. The basic reproduction number (*R*_0_) of COVID-19 was estimated ranging from 2.1 to 5.1 [2]. Asymptomatic SARS-CoV-2 individuals are as infectious as symptomatic ones [3]. Their existence increases the difficulty and uncertainty in pandemic control. Large-scale population testing, contact tracing, quarantine, social distancing, and city lockdown have been implemented in numerous countries and have shown a substantial impact on reducing transmission. However, such a policy has caused severe economic impacts, making it difficult to sustain compliance. In addition to passive defense by avoiding crowd gatherings, one strategy to reduce the infected population is vaccination that provides a high degree of protection in public. The approach to achieving herd immunity has thus been widely proposed, which occurs when a large portion of a population are immune to disease. By doing so, the spread can eventually be stopped. Currently, several types of vaccine have been granted an Emergency Use Authorization (EUA) by the U.S. Food and Drug Administration (FDA) including virus (inactivated vaccines, live-attenuated vaccines), protein subunit, viral vector, and nucleic acid vaccines [4]. Although the effectiveness of vaccines varies, significant protection against COVID-19 has been observed across populations in different countries. Nationwide mass vaccination by the mRNA COVID-19 vaccine in Israel resulted in the reduction of symptomatic COVID-19 cases, hospitalizations, severe illness, and death [5]. The ChAdOx1 nCoV-19 vaccine shows efficacious against SARS-CoV-2 in Brazil, South Africa, and the UK [6]. The results from quarantine, city lockdown and vaccination indicated the need for effective public health strategy to control the pandemic. It is thus critical in implementing the policy according to various scenarios if we are able to predict how fast the spread is, how efficacious the vaccine is, and at what rates the vaccination should be given.

The effectiveness of COVID-19 vaccines raises a potential that city lockdown might not be necessary in the presence of ongoing vaccination, thereby minimizing the economic loss. Here, we considered this possibility by setting up a simulation of COVID-19 outbreak in a small city composed of a highly packed and interacting population of *N* = 2.5 million. Due to economic demands, there was no lockdown or strong spatial restriction in the city. The residents commuted anywhere and daily via public transportation and efficient traffic tools. To prevent the spread of infection, the residents were vaccinated to yield *M* fully vaccinated persons/day, regardless of whether the inoculated person was or had previously been infected. To examine whether the city could prevent the outburst of infection by vaccination, we modeled the progression of the carrier number within a short period (less than one month), during which the majority of carriers remained contagious. In this model, we compared the simulated results in the following setups: the basic reproduction number (*R*_0_) = 1.0–5.5, fully vaccinated rate (*M*) at 10^3^, 10^4^, or 10^5^ persons/day, and the vaccine effectiveness (*v*) at 51% or 89%. Our results indicated that although vaccine inoculation is a very useful tool, policy implementation to reduce the social activity and prevent further infection is a rather urgent approach against COVID-19.

## 2. Materials and Methods

To simulate the dynamics of highly transmissible infectious diseases such as COVID-19, the most commonly used technique is SIR, SEIR, or SIRV compartmental modeling [7,8]. The population is assigned to compartments labeled with (S, I, R), (S, E, I, R), or (S, I, R, V), and ordinary differential equations (ODEs) are used to address the dynamics of these compartments. Specifically, “S” defines the number of individuals “susceptible” to infection. “I” defines the number of “infectious” individuals, including symptomatic and asymptomatic carriers that are able to infect susceptible individuals. “R” stands for the number of individuals “removed” from the infection. “V” represents the number of individuals who have been vaccinated and are resistant to the infection. When a susceptible and an infectious individual come into the transmissible range of infection (through a direct contact or an indirect contact such as droplet- and aerosol-mediated transmission), the susceptible individual can be infected at a certain probability and moved to the “I” compartment. An intermediate “exposed” (“E”) compartment might be assigned if there is a significant latency or delay during which a recently infected individual has not yet become infectious. When the infected individual is recovered and immune to the pathogen, or dies because of the infection, the person is “removed” from the population. Alternatively, if the recovered individual can be re-infected, the person is assigned back to the “S” compartment. More complexity can be added to the model. For example, depending on the nature of infection and the routes of transmission, the “S” compartment can be sub-grouped by parameters such as age, medical condition, and occupations. Likewise, the “I” compartment can be sub-labeled by variables such as the days of infection if the ability to spread the pathogens depends on the progression of the disease. Other variables include the spatial degree of freedom, which is ignored in most compartment models. If spatial constraints (e.g., city lockdown), locality, or flow patterns (e.g., commuting activity) are applied to the community, the compartments might be sub-labeled by spatial degree of freedom and partial differential equations (PDEs) are used to address the dynamics of the compartments.

In this work, we modified the compartmental model by taking into account the following considerations. First, for economic concerns, there was no city restriction law in the community. All residents were allowed to commute anywhere and daily via public transportation and efficient traffic tools. Second, the community had limited resources for fast and accurate COVID-19 screening. A significant portion of the infectious individuals was not identified. As such, we assumed that the susceptible and infectious individuals were well mixed in the community. This assumption allowed us to ignore the spatial degree of freedom and approximate the rate at which the number of infectious individuals increased, *Г*, as *r* × (*S*/*N*) × *I*, where *r* was the rate for an infectious individual to spread the infection on a population with all subjects being susceptible. When the community was partially susceptible, *Г* was justified by multiplying *r* with the susceptibility of the community, *S*/*N*, where *N* was the total number of population. The derivation of this approximation followed the Kermack–McKendrick theory [9] and *r* was related to the basic reproduction number *R*_0_ (i.e., the expected number of secondary infections from a single infection in a population with all subjects being susceptible) [10]. In general, *r* could be estimated by normalizing *R*_0_ with respect to the average recovery time for an infectious individual. Third, the incubation period and the infectious period of COVID-19 vary significantly among infected individuals (1–14 days and 8–10 days, respectively), and the virus could still be detected for 20 days or longer after the initial onset of symptoms [11]. Detection of virus in the fecal samples of patients five weeks after the first onset of symptoms has also been reported [12]. In this regard, for a short-term community outburst, the recovery from the infection could be neglected. We thus ignored the “R” compartment and assumed that all infectious individuals remained contagious throughout the entire course of simulation and that they were infectious once being infected. Fourth, breakthrough COVID-19 infections in fully vaccinated individuals have been reported [13,14,15,16,17]. To include this possibility, we set *v* as the effectiveness (≤1) at which a fully vaccinated individual was protected from the infection and (1 − *v*) as the probability at which the person could be infected. If the fraction of fully vaccinated individuals in the susceptible compartment was set as *q*, the rate *Г* at which the number of infectious individuals increased could be re-justified as *r* × ((1 − *q*) + *q* × (1 − *v*)) × (*S*/*N*) × *I*, where the term *q* × (1 − *v*) indicated the fraction of fully vaccinated individuals the vaccines failed to protect. Fifth, COVID-19 vaccines can take 2–3 weeks from the final vaccination to be fully effective [18]. In our simulation, the vaccinated individuals were set as those who had taken the final vaccination and developed immunity against COVID-19 (despite the fact that they could still be infected and become infectious). Due to the lack of efficient screening, we assumed that the residents were inoculated at a constant rate to yield *M* fully vaccinated persons per day until the entire population was inoculated, regardless of whether the inoculated person was or had previously been infected. In term of the inoculation rate, if there was no vaccine-induced death or fluctuation in the onset of vaccine-mediated immunity, with a consistent daily inoculation of vaccines, the rate at which fully vaccinated individuals were produced should be identical to the rate of inoculation (i.e., in a constant flux). Note that the variation of vaccine effectiveness could be due to the choice of vaccine (i.e., different brands or types) or the protocol of vaccination (i.e., one dose, two doses, etc.). Finally, we assumed that there was no death or birth in the community during the simulation.

Following the modifications and assumptions above, we used a mean-field approach to address the average effects of contagious spreading and vaccinated protection. Since we were considering a short-term outburst of the infection and ignored the recovery of infectious individuals, the two remaining parameters were the spreading rate of the infection and the rate at which the residents were fully vaccinated. We set *N* as the total number of population in the community. Before the simulation started, the community had already had community infection, along with ongoing inoculation of vaccines to yield *M* fully vaccinated persons per day. Day 1 of the simulation was set on the date on which the community had *M* fully vaccinated persons (2*M* on day 2, 3*M* on day 3, etc.). The number of infectious individuals, *I*, was set as *P*(*t*) and the fraction of infectious individuals with respect to the entire population was set as *p*(*t*) = *P*(*t*)/*N*, where *t* was the days in the simulation and *P*(0) = *P*_0_ was the initial number of infectious individuals. The number of susceptible individuals was then *S* = *N − P*(*t*). Since we ignored the recovery from the infection, we had the fraction of susceptible individuals (including those vaccinated) with respect to the entire population, *S*/*N*, as (1 − *p*(*t*)). In addition, we set the number of fully vaccinated individuals as *Q*(*t*) and the fraction of fully vaccinated individuals with respect to the entire population as *q*(*t*) (*q*(0) = 0, *q*(1) = *M*/*N*, *q*(2) = 2*M*/*N*, etc.). Note that this fraction remained identical in both the infectious and susceptible compartments as the inoculation of vaccines was given regardless of whether the receiver was or had previously been infected.

To address breakthrough infections, we assumed that the community used two different types of vaccine (or protocols), one with 51% and another with 89% effectiveness against COVID-19 infection (i.e., *v* = 51% and 89%). Since we were taking a mean-field approach, the spreading rate of infectious individuals, *r*, was estimated by normalizing the average basic reproduction number of the infection, *R*_0_, with respect to the average infectious period *T_i_*. In principle, *r* could be time-dependent due to the changes of public health polices and the way people commuted and interacted in the community. Here, for simplicity, we set it as a constant during the entire simulation.

Using the notations above, we obtained the temporal evolution of the fully vaccinated and the infectious individuals via the following equations:

(1) $$\frac{dQ\left(t\right)}{dt}=M$$

(2) Q(t)=Mt≤N

$$\to q\left(t\right)=\frac{Mt}{N}\;(\leq \;1\;\mathrm{for}\;t\;\leq \;N/M,\;\mathrm{and}\;1\;\mathrm{otherwise}),$$

(3) $$\frac{dP\left(t\right)}{dt}=r\left(t\right)\left\{\left[\left(1-v\right)q\left(t\right)+\left(1-q\left(t\right)\right)\right]\left(1-p\left(t\right)\right)\right\}P\left(t\right)$$

$$\to \frac{dp\left(t\right)}{p\left(t\right)\left(1-p\left(t\right)\right)}=dt\cdot r\left(t\right)\left(1-vq\left(t\right)\right)$$

(4) $$P\left(t\right)=\frac{N}{1+\frac{1-p_{0}}{p_{0}}exp\left[-\int_{0}^{t}d\tau \cdot r\left(\tau \right)\left(1-\frac{vM\tau }{N}\right)\right]}$$

In Equation (3), we justified the spreading rate by multiplying *r* with the remaining fraction of population not effectively protected by the vaccination. Note that in Equation (3), the entire pre-factor in front of *P*(*t*) resembled a growth rate of *P*(*t*) and thus could be considered as the “reproduction rate” of the infectious individuals. What should also be noted is the change of infected rates between the vaccine protected and non-protected individuals. With the fraction of fully vaccinated individuals with respect to the entire population as *q*(*t*) and the effectiveness of full vaccination against the infection as *v* (≤1), the overall susceptibility of the population followed [(1 − *q*(*t*)) + *q*(*t*) × (1 − *v*)] × (*S*/*N*) instead of (*S*/*N*), where *S* = *N* − *P*(*t*). Likewise, with *r* as the spreading rate per infectious individual on a population with all subjects being susceptible, the spreading rate per infectious individual on a fully vaccinated population was then *r* × (1 − *v*) and the overall spreading rate per infectious individual on a partially vaccinated population followed *r* × [(1 − *q*(*t*)) + *q*(*t*) × (1 − *v*)]. The combination of these factors led to the modified “reproduction rate” per infectious individual, *r* × [(1 − *q*(*t*)) + *q*(*t*) × (1 − *v*)] × (*S*/*N*) = *r* × [1 − *q*(*t*) × *v*] × (1 − *p*(*t*)), as shown in Equation (3). When *r*(*t*) did not explicitly depend on time, we had a simple variation of Equation (4) as the following:

(5) $$P\left(t\right)=\frac{N}{1+\frac{1-p_{0}}{p_{0}}exp\left[-\frac{R_{0}}{T_{i}}\left(1-\frac{vM}{2N}t\right)t\right]}$$

## 3. Results

**Figure 1:**
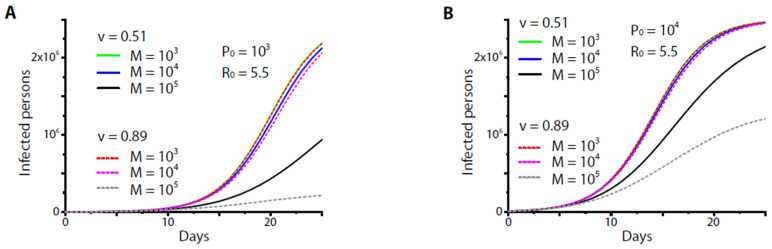
The numerical results from Equation (5) for two different initial carrier numbers *P*_0_ (=10^3^ in (**A**) and 10^4^ in (**B**)) and various vaccination rates to yield *M* fully vaccinated persons/day. Note that curves at *M* = 10^3^–10^4^ persons/day were almost overlapped regardless of the initial condition and vaccine effectiveness. A significant reduction of infected persons occurred only if *M* = 10^5^ persons/day (black and grey lines). For all the results, the average infectious period *T_i_* was set as 14 days, the number of entire population *N* was set as 2.5 × 10^6^, and the basic reproduction number *R*_0_ was set as 5.5. *v* = 0.51 or 0.89 was the effectiveness of the vaccine.

Figure 1 shows the numerical results of Equation (5) at *R*_0_ = 5.5. We found that neither vaccine (*v* = 51% or 89%) could protect the community regardless of how the inoculation rate (to yield *M* = 1000–10,000 fully vaccinated persons/day) was set and how the initial carrier number varied (Figure 1A,B). The high-end vaccine (*v* = 89%) could protect the community only if it was given at an extremely high inoculation rate (to yield *M* > 100,000 persons/day), namely, at a rate by which at least ~4% of the entire population could be inoculated per day, which might be unrealistic given the limited medical resources of the small community. Since *q*(*t*) was the normalization of daily yields of fully vaccinated individuals, *M*, with respect to the population size *N*, the results were applicable to populations of different size. Furthermore, in our setup, the daily yield of fully vaccinated individuals was roughly identical to the daily inoculation rate. When *N* = 23,000,000–25,000,000 (roughly the size of a small country), the minimal inoculation rate required to achieve reasonable control of the disease was found to be 1,000,000 persons/day, a very unrealistic number for a small country of size of 25 million. 

**Figure 2:**
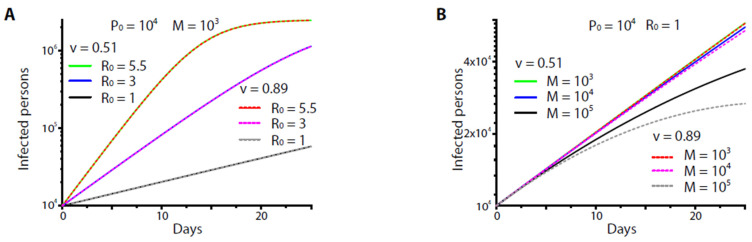
The numerical results from Equation (5) when (**A**) 10^3^ fully vaccinated persons were yielded per day at various *R*_0_, and when (**B**) various fully vaccinated persons (*M*) were yielded per day at *R*_0_ = 1. For (**A**), note that there was almost no difference in the results between the two vaccines and that a significant reduction of infected persons occurred at *R*_0_ = 1 (black and grey lines) For (**B**), note that curves at *M* = 10^3^–10^4^ persons/day were almost overlapped regardless of the vaccine effectiveness. A reduction of the infected persons occurred only when *M* = 10^5^ persons/day (black and grey lines). For all the results, the average infectious period *T_i_* was set as 14 days, the initial carrier number *P*_0_ = 10^4^, and the number of entire population *N* = 2.5 × 10^6^. *v* = 0.51 or 0.89 was the effectiveness of the vaccine.

A dramatic effect occurred, however, if the basic reproduction number *R*_0_ (and hence *r*) was suppressed (Figure 2A). Again, no significant difference was found between the two vaccines (or protocols) if they were given at the moderate, reasonable rates (*M* = 1000–10,000 fully vaccinated persons/day) (Figure 2B). The difference was observed at the extremely high inoculation rate (to yield *M* > 100,000 fully vaccinated persons/day). Nevertheless, it was less than an order of magnitude compared to the high *R*_0_ scenarios (e.g., Figure 1A,B). 

The minimal vaccination rate could also be estimated for a variety of constraints. For example, at present the severity rate and the fatality rate of COVID-19 vary around 0.28–28% among different case studies and reports [19,20]. For a small city, the medical capacity for patients with severe COVID-19 was limited to, say, less than 1000 beds (i.e., at an order of ~0.04% with respect to the entire population). If the severity rate was estimated ~2–3%, such capacity required the number of total carriers by the end of vaccination (i.e., all residents gained fully effective vaccination) to be less than 1% of the entire population. From Equation (2), the time by which all the residents were vaccinated was *T_v_* = *N*/*M*. From Equation (5), the required yield of fully vaccinated persons per day was estimated as (for *P*(*t* = *N*/*M*)/*N* ≤ 1%):

(6) $$M\geq \frac{N\left(1-\frac{v}{2}\right)\frac{R_{0}}{T_{i}}}{ln\left[\frac{\left(1-p_{0}\right)}{99p_{0}}\right]}$$

**Figure 3:**
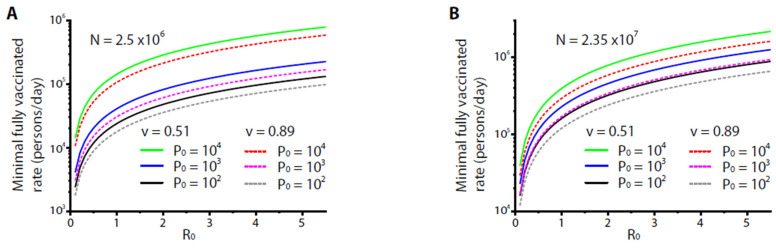
The numerical results from Equation (6) for two different population sizes of *N* (=2.5 × 10^6^ in (**A**) and 2.35 × 10^7^ in (**B**)) and various initial carrier numbers *P*_0_. For all the results, the average infectious period *T_i_* was set as 14 days. *v* = 0.51 or 0.89 was the effectiveness of the vaccine.

Figure 3A shows the numerical results of Equation (6) at *P*_0_ = 100, 1000, and 10,000, under the variation of *R*_0_. The results indicated that the minimal rates to yield fully vaccinated persons per day increased with *P*_0_ and *R*_0_. For *R*_0_ = 5.5, the minimal rates were ~100,000–800,000 fully vaccinated persons per day. Even for the high-end vaccine or protocol (*v* = 89%) with a small number of initial carriers (*P*_0_ = 100), a minimal rate ~100,000 fully vaccinated persons/day was required, which could be a burden for a small city of 2.5 million. Figure 3B scales up the results of Eqn. [6] for a larger community (or a small country) of population *N* = 23.5 million. For *R*_0_ = 5.5, the minimal rates were to yield ~900,000–1,600,000 fully vaccinated persons per day, quite a burden for a small country. For practical purposes, if the rate was set to yield ~100,000 fully vaccinated persons/day, the basic reproduction number *R*_0_ needed to be <0.4, while for a rate to yield ~10,000 fully vaccinated persons/day, *R*_0_ needed to be <0.025. These results indicated the necessity of imposing restrictions such as city lockdown for an effective control of disease by vaccination.

## 4. Discussion

To control disease spreading in an effective way, sustainable strategies should be devised and imposed. Such a scheme requires an understanding of how fast the disease is spreading, how efficacious the vaccine is, and how efficiently the vaccination can be disseminated, thereby prioritizing policy implementation. Data from our modeling estimate the consequences of COVID-19 outbreak in this small city (of a population of 2.5 million). First, we found that at basic reproduction number *R*_0_ = 5.5 neither high vaccination rate (10,000 persons/day) nor high-end vaccine (effectiveness *v* = 89%) could control the disease. Only in the case of extremely high vaccination rates (>4% of the entire population/per day), significant differences in the number of infected people occurred. Scaling this finding to a modest size city, e.g., of a population of 25 million, the effective vaccination rate occurs at 1,000,000 persons per day, which was quite unrealistic. Second, we found that *R*_0_ was critical. Even with a slight reduction of *R*_0_ from 5.5 to 3, a mild vaccination rate (1000 persons/day) was sufficient to suppress the spread of disease. Third and importantly, the vaccine-mediated effect of disease control did not explicitly depend on the choice of vaccines unless the vaccination rate was raised to an unrealistic, extremely high level.

Nevertheless, we should point out that our study had certain limitations as we simplified the dynamics. For example, the effects of vaccination, the ability to spread the virus, and the infectious period possess a certain degree of heterogeneity. In reality, these parameters are not only time-dependent but also depend on the explicit context of how the residents interact and how the city rules are imposed and executed. A more realistic scheme of modeling is to individualize or segregate the carriers and the vaccinated persons into different subgroups, based on their contagious capacity and immune responses under the regulation of city rules for public health. Likewise, we have imposed a mean-field approach to address the infectious period throughout the entire outburst. This approach is oversimplified; yet it provides a quantitative estimate for our purposes. Finally, we have ignored the recovery term in the modeling due to the consideration that we were interested in the dynamics within a relatively short period of outburst. The recovery term certainly has impacts on the dynamics. However, it also possesses a large degree of heterogeneity and can thus make our analyses complicated, which is not our primary purpose. To improve the modeling, more real-world data are necessary to impose into the model and justify its predictions.

For economic concerns, most governments are keen on purchasing high-end vaccine or increasing the vaccination rate to reduce the necessity of city lockdown. Our results, however, highlighted the importance of understanding the transmission dynamics of COVID-19 in determining public health policy. Population size/density, infectious period, and frequency of social contact, which influence the spread of virus, cause a dynamic change of transmission. In light of the fact that *R*_0_ is a very important factor that has been dramatically linked to the scale of disease, our studies indicated essential policy implementation to reduce *R*_0_ as a reasonable and urgent approach for government against COVID-19.

## 5. Conclusions

To have an effective control of COVID-19 in a modest city, it is not recommended to rely on the increase of vaccine inoculation rate only. This study indicated that combination with policy interventions to mitigate community transmission is required, wherein the long-term value of vaccination can occur to protect the community. To gain more insights into these issues, future studies are needed to incorporate more real-world data and more degrees of freedom into the methodology adopted in the present work.

## Data Availability

All the parameters and equations used to generate the results were included in the Materials and Methods and in the Figure captions.
